# Prognostic Factors in Hodgkin Lymphoma

**DOI:** 10.4084/MJHID.2014.053

**Published:** 2014-07-05

**Authors:** Annarosa Cuccaro, Francesca Bartolomei, Elisa Cupelli, Eugenio Galli, Manuela Giachelia, Stefan Hohaus

**Affiliations:** Institute of Hematology, Catholic University S. Cuore, Rome, Italy

## Abstract

Hodgkin lymphoma (HL) is among the neoplastic diseases that has the best long-term outcome after cytotoxic treatment. Cure rates approach 80–90%; however, 15–20% of patients will be resistant to therapy (primary refractory) or relapse after treatment. Prognostic factors should help to stratify treatment according to the risk profile and identify patients at risk for failure. Significance of prognostic factors partly depends on the efficacy of the treatments administered, since new effective therapies can variably counterbalance the adverse effects of some unfavorable clinical determinants. As a consequence, some prognostic factors thought to be important in the past may become meaningless when modern successful therapies are used. Therefore, the value of prognostic factors has to be updated periodically, and then adapted to new emerging biomarkers. Besides the prognostic role of PET imaging, tissue and circulating biomarkers, as the number of tumor-infiltrating macrophages, cytokine and chemokine levels and profiling of circulating nucleic acids (DNA and microRNAs) have shown promise.

## Introduction

Treatment of Hodgkin lymphoma (HL) is an indubitable one of the greatest success stories of medical oncology in the 20th century. Cure rates approach 80–90% of patients, and HL is among the neoplastic diseases that have the best long-term outcome after cytotoxic treatment. However, 15–20% of patients will be resistant to therapy (primary refractory) or relapse after treatment, usually in the first two years. This review will analyze the prognostic factors that can identify patients at risk. Since outcome of patients is determined not only by disease characteristics but also by the risk of short- and long-term sequelae of the treatment, which can even outnumber the events of disease recurrence, the identification of risk factors for secondary events will be increasingly important to tailor the therapy and thus avoiding potential harmful treatments in individuals at risk.

**Figure 1:**
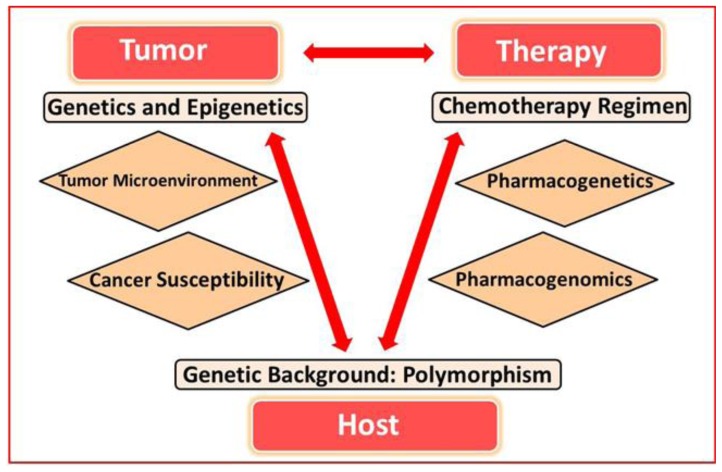
Prognostic factors can be divided into areas that are related to the disease, factors related to the patient as a host for the disease and to the therapy. Interactions between these areas exist. The genetic background of the patient is a host factor that modulates the metabolism of cytotoxic drugs, and as a consequence alters the response and side effects of the treatment.

In a simplified way, prognostic factors can be divided into areas that are related to the disease, factors related to the patient as a host for the disease and to the therapy (Figure 1). Interactions between these areas exist. The genetic background of the patient is a host factor that modulates the metabolism of cytotoxic drugs, and as a consequence alters the response and side effects of the treatment.

In this review, we will only briefly discuss the prognostic relevance of pathological and immunological features of HL, and not consider PET imaging, that has evolved into the most exciting tool to evaluate the prognosis in HL in recent years. This topic will be covered with another review in this issue of the journal. Many prognostic factors, used in standard clinical practice, have been known for a long time. These factors often reflect disease burden and disease activity that is related to the inflammatory microenvironment. Biomarkers described in recent years are indicators of the disease activity as well, but they describe this activity in a more sophisticated, accurate and pathogenetically more relevant way. Often these new prognostic factors still need validation, but they may eventually substitute for classical clinical factors.^1^

## Tumor Burden: Stage and Bulk

**Figure 2:**
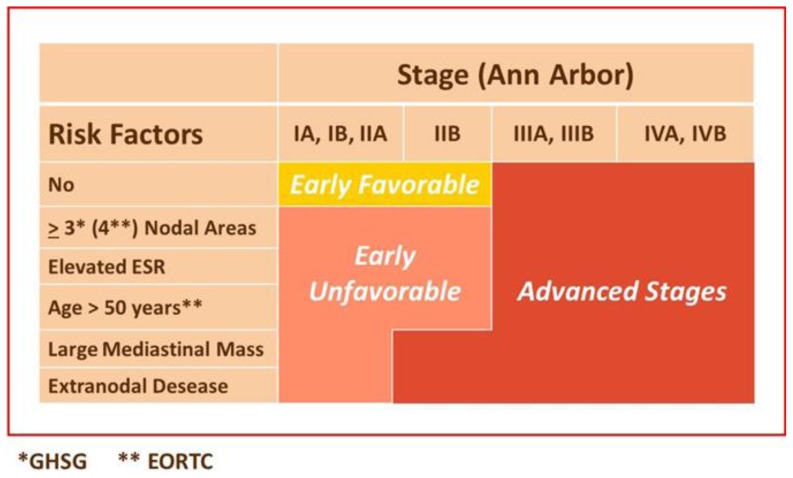
Risk factors in the European Organization for Research and Treatment of Cancer/Groupe d’étude des Lymphomes de l’adulte (EORTC/GELA) and the German Hodgkin Study Group (GSHG) stratification scores for HL.

Extension of disease and tumor burden is indubitable the most important disease characteristic, that is used to stratify treatment strategies (Figure 2). Staging according to the Ann Arbor system is part of clinical routine for more than 40 years.^2^ In limited stage disease, the presence of bulky disease detected on chest radiography or CT at staging is considered a negative predictor of outcome. The presence of a bulky tumor is one of the risk factors in the European Organization for Research and Treatment of Cancer/Groupe d’étude des Lymphomes de l’adulte (EORTC/GELA) and the German Hodgkin Study Group (GSHG) stratification scores for HL.^3^ By contrast, in advanced stage disease, the presence of a bulky tumor is not a risk factor in the international prognostic score (IPS) for HL.^4^ Since measurement of bulk is limited to the single largest mass, it could underestimate the total tumor burden in patients with diffuse disease. Newer methods to measure tumor burden with CT volume or metabolic tumor volume may give more precise estimation of the tumor volume.^5^–^7^ Normalization of the tumor mass to the body surface (the relative tumor burden) yields a parameter with a reliable prediction for tumor control modulated by the use of chemotherapy regimens with different intensity.^7^ The complexity of the evaluation of all lesions in any scan slice with subtraction of normal structures that are present in the tumor tissue, and approximation for bone marrow involvement has limited a wider application of this type of evaluation. Therefore, an indirect estimate was derived from a few staging parameters and demonstrated sufficient statistical reliability when compared with the direct measure of rTB.^8^ The equation {Estimated rTB = −4.3 + 8.3 × IPI2+ 22.7 × [number of involved sites (+3 if bulky mass is present)]} was recently proposed for investigational and clinical uses when the direct measurement cannot be performed.)}]

Spread of HL beyond its lymph node microenvironment to extralymphatic organs is associated with inferior outcome. In limited stage disease involvement of an extranodal site is defined as a risk factor by the GSHG scoring system. In patients with advanced-stage disease, diffuse organ involvement defining stage IV disease is an independent risk factor in the IPS.^9^

## Age

Age is the most important factor when overall survival is analyzed, and remains an independent factor also for progression-free survival. It impacts on prognosis in at least two ways: On one hand, it is intrinsically associated with HL biology and, on the other hand, older age often is associated with co-morbidity and reduced tolerability of chemotherapeutic regimens used in younger patients. HL epidemiology is characterized by a bimodal age distribution. Following the peak in young adults in their twenties, there is a second increase in the incidence, in particular in males, after the age of 50–55 years. When compared to other hematological neoplastic diseases, that usually set the cut-point to define elderly patients at 60 years, the prognostic cut-point in HL is shifted versus a younger age.

In the International Prognostic Score for patients with advanced stage disease the cut-point is age of 45 years, the EORTC lists age more than 50 years as a risk factor for patients with limited stage disease. Older age associates with a higher frequency of the mixed cellularity histotype and presence of EBV in the neoplastic cells, when compared to younger patients.^10^ EBV-association appears to be a prognostic factor that is limited to the elderly patients.^11^–^13^ It is hypothesized that loss of immunological control of EBV-infected cells might contribute to the development of EBV-associated HL in the elderly. Aging of the immune system (immunosenescence) is characterized by reduced function of the adaptive immune response that includes T and B cell function. Studies are required to address the question whether immunosenescence is a mechanism in the pathogenesis of elderly HL, and whether this will contribute to the negative effect of age on prognosis.

Therapy of HL in the elderly is often complicated by toxic side effects of chemotherapy. Standard treatment with ABVD is often not recommended for patients older than 70 years. Bleomycin leads to frequent incidence of pulmonary toxicity in the elderly. In a recent report, the incidence of bleomycin lung toxicity was 32% with a 25% mortality.^14^ Intensified regimens as the BEACOPP-dose escalated regimen are not recommended for patients with advanced-stage HL over 60 years.^15^ However, even in patients over 50 years with reduced performance status, mortality of BEACOPP-dose escalated increases to 13.3%.^16^ Therapy of elderly patients with HL remains a challenge, and effective regimen with acceptable toxicity profiles is still lacking. The availability of antibody-drug conjugates, as Brentuximab may be major step forward.

## Gender

Males with HL have a poorer outcome than females. This effect of gender is not limited to HL. As well, female patients with follicular lymphoma and diffuse large B cell lymphoma fare better than their male counterparts. On the mechanism of the gender effect on prognosis in HL, one can only speculate, but it could influence prognosis in at least two ways. A preponderance of male gender is observed in elderly patients, and as a consequence males have more often unfavorable disease characteristics. Another mechanism for the gender effect in lymphoma may be due to differences in pharmacokinetics. Female patient with HL experiences more hematological toxicity, especially more severe leucopenia, probably due to gender difference in metabolism of cytotoxic drugs of the ABVD regimen.^17^ Moreover, hematological toxicity has been associated with a more favorable outcome.

## B-Symptoms

Constitutional symptoms defined by unexplained fever >38°C, drenching night sweats and weight loss >10% of the weight are a presenting sign in about 10–25% of patients with limited stage disease, and up to 70% of patients with advanced stage disease.^9^ Among the symptoms, isolated night sweats do not appear to be associated with inferior outcome. The presence of B-symptoms is a risk factor, in particular in stage II bulky disease, that is not considered to be a limited stage disease by the German Hodgkin study group when B-symptoms are present.

B-symptoms are due to the production of pro-inflammatory cytokines by the Hodgkin tumor tissue, in particular IL-1, TNF-alpha, and IL-6.^1^ B-symptoms are associated with a variety of other laboratory abnormalities and patients characteristics, and in multivariate analyses it has therefore often been removed in final models, as in the IPS.

## Anemia

Anemia is a frequent finding at HL diagnosis and is present in about 40% of patients. It is usually a mild to moderate normocytic anemia, with the characteristics of anemia of inflammation. Cut-off point for prognosis in the IPS is a hemoglobin level of 10.5 g/dl, and this is independent of gender.

We demonstrated that elevated IL-6 levels correlate with hemoglobin levels and that IL-6 levels correlate with levels of hepcidin, an acute phase reactant and a major regulator of iron metabolism.^18^ Therefore, anemia is linked to the inflammatory activity of the HL microenvironment, and this might explain its big prognostic impact.

Anemia of inflammation is characterized by alterations in iron metabolism. Elevated production of hepcidin blocks the release of iron from the intestine and iron stores in the reticuloendothelial system that results in increasing levels of ferritin. Elevated ferritin levels have been described in HL and have been associated to prognosis four decades ago.^19^ The accumulation of iron also in the HL microenvironment can have biologic effects on cell function and induce cell damage by induction of reactive oxygen species (ROS) that interfere with the function of macromolecules as DNA, and proteins.

## The White Blood Cells: Leukocytosis, Lymphopenia, Monocytosis

Alterations of the counts and composition of the white blood cells in peripheral blood are often at diagnosis in HL and well known prognostic factors. Typical alterations in WBC counts include leukocytosis with neutrophilia, lymphocytopenia, either relative or absolute, and monocytosis.

In the IPS the prognostic cut point for white blood cell count is set at 15000/microL, for lymphocytopenia it is 600/microL or less than 8%..^9^ More recently, the monocyte count, in particular in relation to the lymphocyte count has been reported to be a prognostic factor in HL and other lymphomas.^20^ In a case cohort of 474 patients with HL observed from 1974 to 2010, monocyte count of >900/microL was associated with inferior progression-free and overall survival. The impact of the monocyte counts on prognosis became particularly evident when the ratio between lymphocytes and monocytes (ALM ratio) was < 1.1. As the number of macrophages in the HL tissue is strongly associated with prognosis, the question arises whether the number of monocytes in PB and the number of tumor-associated macrophages (TAM) are correlated.

## Albumin

Low levels of serum albumin are associated with a worse prognosis in many hematological neoplasias, including HL. The IPS score defines albumin levels of 4.0 g/dl as cut-point. Albumin is produced by the liver, and about 12–20% of the protein synthesis capacity of the liver is dedicated to albumin production. Albumin synthesis is reduced when synthesis of acute-phase proteins is stimulated by IL-6 or when availability of amino acids is decreased due to reduced nutritional status. Albumin levels inversely correlate to IL-6, TNF alpha and IL1-RA.^1^

## The Erythrocyte Sedimentation Rate

The erythrocyte sedimentation rate, albeit its nonspecific character is one of the oldest prognostic factors for HL. It is still in use to define early stage HL as favorable or unfavorable. The EORTC and GSHG set the cut-point to 30 mm/h for patients with B –Symptoms and 50 mm/h for patients without B-symptoms. The ESR is increased in many diseases, in particular in those with an inflammatory reaction. The ESR can be altered by many variables, as the erythrocyte count and the protein composition in the plasma, in particular increased levels of fibrinogen, acute phase proteins and gamma globulins can increase the ESR. As these parameters are as well prognostic markers in HL, the ESR does often not maintain its value in multivariate analysis.

## Beta2-microglobulin

B2M is a component of the HLA-I antigen and present on the surface of nearly all nucleated cells in the body. In healthy people, it is produced at a constant rate and eliminated in the kidney where free glomerular filtration is followed by tubular re-adsorption. Lymphocytes are the main production site of b2M, and inflammatory cytokines stimulate the production of b2M, and increased levels of b2M can be due to increased release from immune system activation or proliferation or decreased renal clearance. It is a prognostic marker in many lymphomas, including HL. Elevated levels of B2M can be found in 5–30% of patients at diagnosis, depend on the stage, and have been found to be associated with the relapse.^21^–^23^

## Biohumoral Factors: IL-10, IL-6, sCD30, TNF, TARC

A large array of cytokines can be detected at increased levels in peripheral blood in HL. These are produced both by the HRS cells and the surrounding microenvironment. The prognostic significance of cytokine levels has ben studied for more than 20 years in HL, and the most frequently studied cytokines are IL-10, IL-6, TNF alpha and its soluble receptors, and more recently the chemokine TARC. IL-10 is of particular interest in the immunopathogenesis of HL, as it is supposed to play an important role in the shift of T cell function from Th1 to Th2 and Treg functional state. IL-10 levels are elevated in about 40–50% of patients, and associate with inferior outcome.^1^,^24^–^30^ IL-10 levels appear to be higher in EBV-associated HL.^29^

IL-6 is a pro-inflammatory cytokine that is associated with some clinical and laboratory manifestations of HL, as, B-symptoms, anemia, and low albumin levels.^31^ IL-6 can induce the production of hepcidin, a major regulator of iron metabolism and mediator for anemia of inflammation or chronic disease, characterized by iron-restriction. We showed that IL-6 – hepcidin axis is active in anemia associated with HL, but that other IL-6-induced, hepcidin-independent mechanisms probably play a role.^30^

The circulating CD30 antigen sCD30, is thought to be shed form the CD30+ HRS cells, and represents, therefore, at least theoretically, an ideal tumor marker for the neoplastic cells. sCD30 levels are increased in about 25–30 % of patients with HL, and, levels above 100–200 U/ml associate with worse outcome.^32^

Ma et al.^33^ used a proteomic approach to screen for proteins in plasma at HL diagnosis to identify new protein biomarkers. The most promising biomarkers appeared to be TARC (thymus and activation-regulated chemokine), a chemokine that is important for attracting immune cells with specific functions to the microenvironment.

The chemokine TARC has recently attracted more interest as it plays a central role in the composition of the microenvironment attracting Th2 and Treg cells. TARC levels are elevated in the vast majority of patients with HL at diagnosis, and rapidly turn to normal during treatment.^34^–^35^ Preliminary data indicate an association between changes in TARC concentration in plasma and therapy outcome. Whether this early change can be a marker to evaluate response has to be addressed in larger studies.

Casanovas and colleagues developed a prognostic score based on different cytokine levels.^1^ The score included IL-6, sCD30 and TNFR1 and was more predictive than standard clinical score. While this work is of high interest, these data need confirmation on independent data sets and in relation to the results of early or interim PET.

## Prognostic Relevance of Characteristics of HRS Cells

The number and atypia of HRS cells together with the degree of cellularity in the nodules and the amount of sclerosis are the characteristics for the separation of nodular sclerosis (NS) into grade 1 and grade 2 according to the British National Lymphoma Investigation (BNLI)[].^36^ NS grade 2 typically is more aggressive, and has an inferior outcome. However, difficulties to reproduce this classification has resulted in conflicting data and limited the widespread use of this classification.^37^–^38^

Several studies indicated that BCL-2 expression in HRS cells is associated with an inferior prognosis.^39^–^41^ However, the relationship between BCL-2 expression and patient outcome in HL remains controversial because other studies have not demonstrated the same correlation between bcl-2 expression and failure-free survival.^42^ Similarly, the association of p53 with patient outcomes in HL remains controversial^40^–^42^ although more studies suggest a prognostic role for BCL-2 than for p53.

## Prognostic Relevance of the Tumor Microenvironment

HL is characterized by an expansion of T cells with a T helper2 and T regulatory phenotype in the microenvironment. However, both immunohistochemistry and gene expression studies indicate that high numbers of T cells with a cytotoxic phenotype and low numbers of FOXP-3 + T reg cells in the microenvironment are associated with inferior outcome.^43^–^45^ A number of other components in the microenvironment as, B cells and eosinophils have been reported to be associated with prognosis.^46^–^47^ However, this information is not part of the routine evaluation for the prognostic purpose.

A more recent tissue biomarker is the number of tumor-infiltrating macrophages, identified by immunohistochemical staining for the CD68 antigen,^48^ which is a relatively simple tissue biomarker of gaining widespread interest.^49^ However, not all studies could confirm the prognostic impact of the count of tumor-associated macrophages in HL. Further studies are needed to determine the optimal antigen (e.g. CD68 versus CD163), anti-CD68 antibody clone (e.g. KP1 versus PGM1) and scoring thresholds (e.g. manual versus computer-assisted) for detecting HL associated macrophages.^49^ The Vancouver group developed a 23-gene outcome predictor that was superior to the IPS and to CD68 immunohistochemistry.

## Circulating DNA of Cellular and Viral (EBV) Origin

Cell-free DNA of cellular and viral origin can be detected in the plasma of patients with HL at diagnosis.^50^–^51^ Cell-free DNA is released from the tumor tissue, and levels correlate to disease activity.^52^ Cell-free DNA is probably released both by the tumor cells and the surrounding microenvironment. The identification of recurrent mutations in patients with HL opens the possibility to develop sensitive techniques to detect these mutations in the cell-free DNA fraction as specific tumor markers in the peripheral blood.^52^–^53^ Patients with cell-free DNA levels above the normal range have an inferior event-free survival.^50^ In the same line, EBV-DNA can be detected in the plasma of patients with HL, and represents a marker for the activity of EBV-associated HL.^54^ It is important to underline that detection of EBV in plasma, but not in the mononuclear cell fraction is associated with the EBV-status in HL.^51^,^54^ We and others have shown that the presence and level of EBVDNA is a prognostic marker.^51^,^54^–^55^

## Genetic Background

Genome-wide association studies (GWAS) on large cohorts of patients with HL have defined the role of polymorphic germ line variants as a risk factor for the development of HL.^56^–^58^ These studies commonly identified a locus on chromosome 6 in the HLA region as a highly significant risk allele for HL. Other single nucleotide polymorphisms, in other HLA regions and cytokine genes, as IL-13 have also been associated with HL risk. About the role of the genetic background as a factor that can modulate the response to treatment and outcome of patients with HL, no results of GWAS are available. Using a target gene approache, we and others reported on the prognostic impact of SNPs in HL. Most of genes studied were involved in the metabolism of cytotoxic drugs, as detoxification enzymes, and immunoregulatory genes, given the pivotal role of immunological alterations and interactions in the pathology of the disease.^59^–^66^ In particular, we found deletions of GSTT1 and GSTM1 and a variant in the GSTP1 gene (Val105Ile), which reduces the enzymatic activity, be associated to a better outcome.^59^–^60^ These data could only be partially confirmed on an independent cohort of patients with advanced HL included in multicenter trial.^67^ Other studies reported on the prognostic impact of genes coding for enzymes involved in drug metabolism are and UGT1A1 and GSTA1.^61^,^64^ We reported that carriers of variants in the promoter region of the IL-6 and IL-10 genes that are supposed to influence gene expression were associated with prognosis in HL.^68^ Validation on independent and large patient cohorts is needed before the germ line variants in the genetic background can be clinically used to modulate the treatment.

## Prognostic Scores

Clinical and laboratory parameters have been combined into different prognostic scoring systems. Patients with limited stage disease are traditionally divided into a favorable/unfavorable group according to the presence of risk factors defined with some minor differences by the GSHG and EORTC (Figure 2).^69^ Both GSHG and EORTC use this classification to design treatment protocols that adapt therapy intensity according to the risk group.

Hasenclever et al. used the data on 5141 patients with advanced stage disease treated with ABVD or COPP-ABVD to develop a 7-point score (international prognostic score, IPS). In order to stratify patients with advanced stage disease, patients are divided into two risk groups (IPS 0–2 vs. IPS 3–5). However, stratification of therapy according to Hasenclever score has not entered clinical routine.

Risk scores have also been developed for patients with relapsed disease. Risk factors as early relapse within 12 months, presence of B-symptoms and extranodal disease are the most important clinical factors, as anemia appears to be the most significant laboratory anomaly to predict poor outcome in relapsed patients.^67^,^69^

## Conclusions

In conclusion, a plethora of prognostic factors is available in HL. Traditional clinical and laboratory prognostic factors often represent a surrogate marker for biological characteristics that often are not included in the standard evaluation. There is no current consensus on how to integrate these biological markers with accepted clinical prognostic risk factors into prognostic scores or how to use this information to adapt treatment. It remains a challenge to identify the best parameters to predict prognosis in the single patient and identify the still significant group of patients for whom standard treatment is not sufficient.
